# The risk for subsequent primary lung cancer after cervical carcinoma: A quantitative analysis based on 864,627 cases

**DOI:** 10.1371/journal.pone.0305670

**Published:** 2024-06-24

**Authors:** Sheng Gong, Gang Li, Dan Li, Yu Liu, Banggui Wu

**Affiliations:** Department of Thoracic Surgery, The Public Health Clinical Center of Chengdu, Chengdu, P.R. China; West China Hospital of Sichuan University, CHINA

## Abstract

**Purpose:**

To compare the risk of developing subsequent primary lung cancer among cervical cancer patients and the general population.

**Methods:**

Several databases were searched from inception to April 25, 2023. The standard incidence ratios (SIRs) with 95% confidence intervals (CIs) were combined to identify the risk for second primary lung cancer after cervical carcinoma. Subgroup analyses based on the follow-up period, age, degree of malignancy and source of SIR were conducted. All the statistical analyses were performed with STATA 15.0 software.

**Results:**

A total of 22 retrospective studies involving 864,627 participants were included. The pooled results demonstrated that cervical cancer patients had a significantly greater risk for lung cancer than did the general population (SIR = 2.63, 95% CI: 2.37–2.91, P<0.001). Furthermore, subgroup analyses stratified by follow-up period (<5 years and ≥5 years), age (≤50 years and <50 years), and degree of malignancy (invasive and in situ) also revealed an increased risk of developing lung cancer among cervical carcinoma patients.

**Conclusion:**

Cervical cancer patients are more likely to develop subsequent primary lung cancer than the general population, regardless of age, follow-up time or degree of malignancy. However, more high-quality prospective studies are still needed to verify our findings.

## Introduction

According to the most recent cancer epidemiology data, 14,100 new cases of uterine cervix disease and 4,280 related deaths are estimated to occur in the United States in 2022. Although the human papillomavirus (HPV) vaccine has gained popularity in recent years, the incidence rate of cervical carcinoma is still relatively high, and it remains one of the causes of tumor-related deaths among women [1]. However, the mortality rate of cervical cancer patients has been declining in recent decades due to great efforts in screening techniques and awareness and improvements in treatment strategies [2, 3]. Thus, the number of patients with a history of cervical carcinoma has increased significantly in the clinic.

Lung cancer remains the second most common malignancy and the leading cause of cancer-related deaths among women in the United States, with 118,830 estimated new cases and 61,360 related deaths in 2022 [1]. Moreover, an obvious increasing trend in the incidence of lung cancer in young women was observed in the past decade [4–6]. Therefore, it is believed that there are more lung cancer patients with previous cervical cancer than patients with previous cervical cancer.

Cancer survivors often suffer from long-term “side effects” caused by the disease and its treatment and are more likely to develop new primary cancers in most cases [7]. There are several potential reasons for this phenomenon. First, chemoradiotherapy, a common antitumor treatment, can cause DNA damage, cell death and a disturbed immune microenvironment [8]. Second, cancer patients may have poorer nutritional status, which reduces the body’s ability to prevent tumorigenesis [9]. Third, patients with cancer are more likely to receive a physical examination, which contributes to screening for early-stage tumors.

According to previous literature, cervical cancer patients are reported to have an 8% increased risk for second primary cancers, especially radiation site-associated tumors and smoking-related tumors such as vagina, vulva, pulmonary and esophageal cancers [10]. For lung cancer, it has been reported that the occurrence of lung cancer is also significantly associated with HPV infection [11, 12]. Considering these common risk factors, including HPV infection, smoking status and female hormones, cervical cancer patients are suggested to have a greater risk of lung cancer than the general population. However, it has also been reported that some factors, such as chemotherapy, may have a protective effect on second primary cancer patients [13]. Thus, it is still necessary to further identify the risk of developing subsequent primary lung cancer among cervical cancer patients, which might help with the clinical management of patients with previous cervical carcinoma.

The aim of this meta-analysis was to identify the risk for subsequent primary lung cancer in cervical cancer patients based on current evidence and relevant data.

## Materials and methods

This meta-analysis was conducted according to the Preferred Reporting Items for Systematic Reviews and Meta-Analyses guidelines (2020) [14].

### Literature search

The PubMed, EMBASE, Cochrane Library and Web of Science electronic databases were searched from their inception to April 25, 2023. The following terms were used during the search: cervical cancer, cervical carcinoma, carcinoma of the cervix, lung, pulmonary, cancer, tumor, carcinoma, neoplasm, subsequent primary and second primary. The detailed search strategy for PubMed was as follows: (cervical cancer OR cervical carcinoma OR carcinoma of cervix) AND (lung OR pulmonary) AND (cancer OR tumor OR carcinoma OR neoplasm) AND (subsequent primary OR second primary). Moreover, free words and MeSH terms were applied, and references cited in the included publications were also reviewed.

### Inclusion and exclusion criteria

The inclusion criteria were as follows: 1) patients who were pathologically diagnosed with primary cervical cancer; 2) patients whose subsequent primary lung cancer was also pathologically diagnosed; 3) patients whose standard incidence ratio (SIR) with 95% confidence interval (CI) was calculated by comparing the incidence rates of lung cancer in cervical cancer patients and the general population; 4) patients whose SIRs with corresponding 95% CIs were reported or for whom enough data were available for calculation; and 5) patients whose full texts were available.

The exclusion criteria were as follows: 1) duplicated or severely overlapping data (>50%); 2) meeting abstracts, letters, editorials, animal trials or case reports; 3) small sample sizes with < 1000 cases; and 4) insufficient data to calculate SIRs with 95% CIs.

### Data extraction and quality assessment

The following information was collected from the included studies: the name of the first author, publication year, sample size, source of participants (region or database), year of diagnosis, follow-up time, age, degree of cervical cancer malignancy, source of SIRs, SIRs and 95% CIs.

The quality of the included studies was assessed according to the Newcastle Ottawa Scale (NOS) score, and studies with an NOS score of 6 or higher were regarded as high-quality studies [15].

The literature search, selection, data extraction and quality assessment were independently performed by two authors (Sheng Gong and Gang Li).

### Statistical analysis

All the statistical analyses were conducted with STATA 15.0 software. The SIRs and 95% CIs were combined to identify the risk for subsequent primary lung cancer among cervical cancer patients compared to the general population. When the SIRs and 95% CIs were not provided, they were calculated based on the number of observed and calculated cases among the cervical carcinoma patients and the general population. The heterogeneity among the included studies was assessed by I^2^ statistics and Q tests. When significant heterogeneity was detected and presented as I2>50% or P<0.1, the random effects model was applied; otherwise, the fixed effects model was used [16]. In addition, subgroup analyses stratified by the follow-up period (<5 years and ≥5 years), age (≤50 years old and <50 years old), and degree of malignancy (invasive and in situ) were conducted to identify the source of heterogeneity and stability of the pooled results in this meta-analysis. Furthermore, Begg’s funnel plot and Egger’s test were conducted to detect publication bias [13, 17]. A P value<0.05 was regarded as a significant difference.

## Results

### Literature search and selection

**Fig 1:**
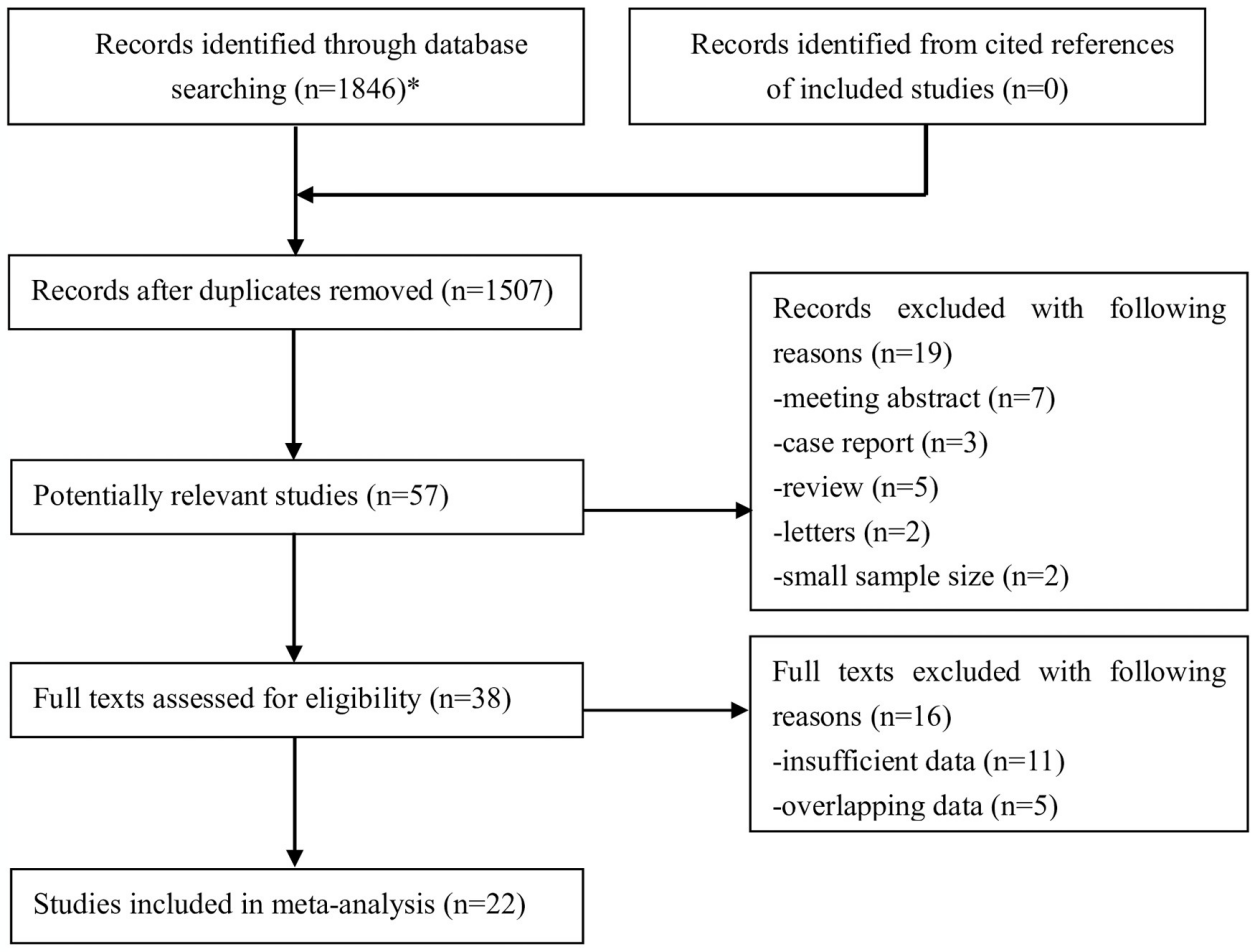
The flow diagram of this meta-analysis. *PubMed (n = 783), EMBASE (n = 56), Cochrane Library (n = 338) and Web of Science (n = 669).

Initially, 1,846 records were found in these four electronic databases, and 339 duplicated records were removed. After screening the titles and abstracts, 1,450 publications were excluded, and 19 records were excluded among the remaining 57 publications. Thus, 38 full texts were carefully reviewed for eligibility, and 22 retrospective studies were eventually included [10, 18–38]. The specific literature selection process is shown in Fig 1.

### Basic characteristics of the included studies

The 22 studies were published between 1974 and 2020, and the sample sizes ranged from 763 to 182,040, with a total of 864,627 patients. Patients were diagnosed between 1935 and 2011. All included studies were high-quality studies with an NOS score of 6 or higher. The other detailed data are presented in Table 1.

**Table 1:** Basic characteristics of included studies.

Author	Publication Year	Sample size	Region or database	Diagnosis year	Source of SIR	NOS
Newell [18]	1974	4871	CHLTR	NR	E	6
Kapp [19]	1982	763	YUMC	1953–1972	E	6
Clarke [20]	1984	7535	OCTRF and OCI	1960–1975	E	7
Boice [21]	1985	182040	15 cancer registries	1960–1970	E	7
Storm [22]	1988	44440	DCR	1943–1982	E	7
Arai [23]	1991	11855	NIRS, NCCH, CIH, SUH	1961–1981	E	7
Rabkin [24]	1992	25295	CTR	1935–1988	R	7
Levi [25]	1993	34615	VCR	1974–1989	E	7
Bergfeldt [26]	1995	5325	SGCR	1958–1992	R	7
Bjorge [27]	1995	37001	Norway	1990–1992	R	7
Kleinerman [28]	1995	86193	13 cancer registries from 5 countries	1935–1990	R	7
Mccredie [29]	1996	6694	New South Wales [Australia]	1972–1991	R	7
Fisher [30]	1997	4457	MTR	1985–1987	R	7
Hemminki [31]	2000	135386	SFCD	1958–1996	R	8
Evans [32]	2003	21605	China	1960–1999	R	7
Taylor [33]	2006	56020	CCR	1988–1999	R	7
Berrington de Gonzalez [34]	2011	19273	SEER	1973–2002	E	8
Chen [35]	2012	52972	TCR**	1979–2008	R	7
Arnold [36]	2014	12048	NCR	1989–2008	R	6
Lim [10]	2016	72805	KCCR	1993–2010	R	7
Bright [37]	2019	23281	ONS, WCR	1971–2006	R	7
Sung [38]	2020	20153	SEER	1992–2011	R	8

CHLTR: Charity Hospital of Lourisiana Tumor Registry; YUMC: Yale University Medical Center; OCTRF: Ontario Cancer Treatment and Research Foundation; OCI: Ontario Cancer Institute; DCR Danish Cancer Registry; NIRS: National Institute of Radiological Sciences Hospital; NCCH: National Cancer Center Hospital; CIH: Cancer Institute Hospital; SUH: Shinshu University Hospital; CTR: Connecticut Tumor Registry; VCR: Vaud Cancer Registry; SGCR: Stockholm-Gotland Cancer Register; MTR: Michigan Tumor Registry; SFCD: Swedish Family-Cancer Database; TCR*: Thames Cancer Registry; CCR: California Cancer Registry; SEER: Surveillance, Epidemiology, and End Results; NCR: Netherlands Cancer Registry; KCCR: Korea Central Cancer Registry; ONS: Office for National Statistics; WCR: Welsh Cancer registry; NR: not reported; SIR: standard incidence ratio; E: estimated; R: reported; NOS: Newcastle Ottawa scale.

### The risk for subsequent primary lung cancer after cervical cancer

**Fig 2:**
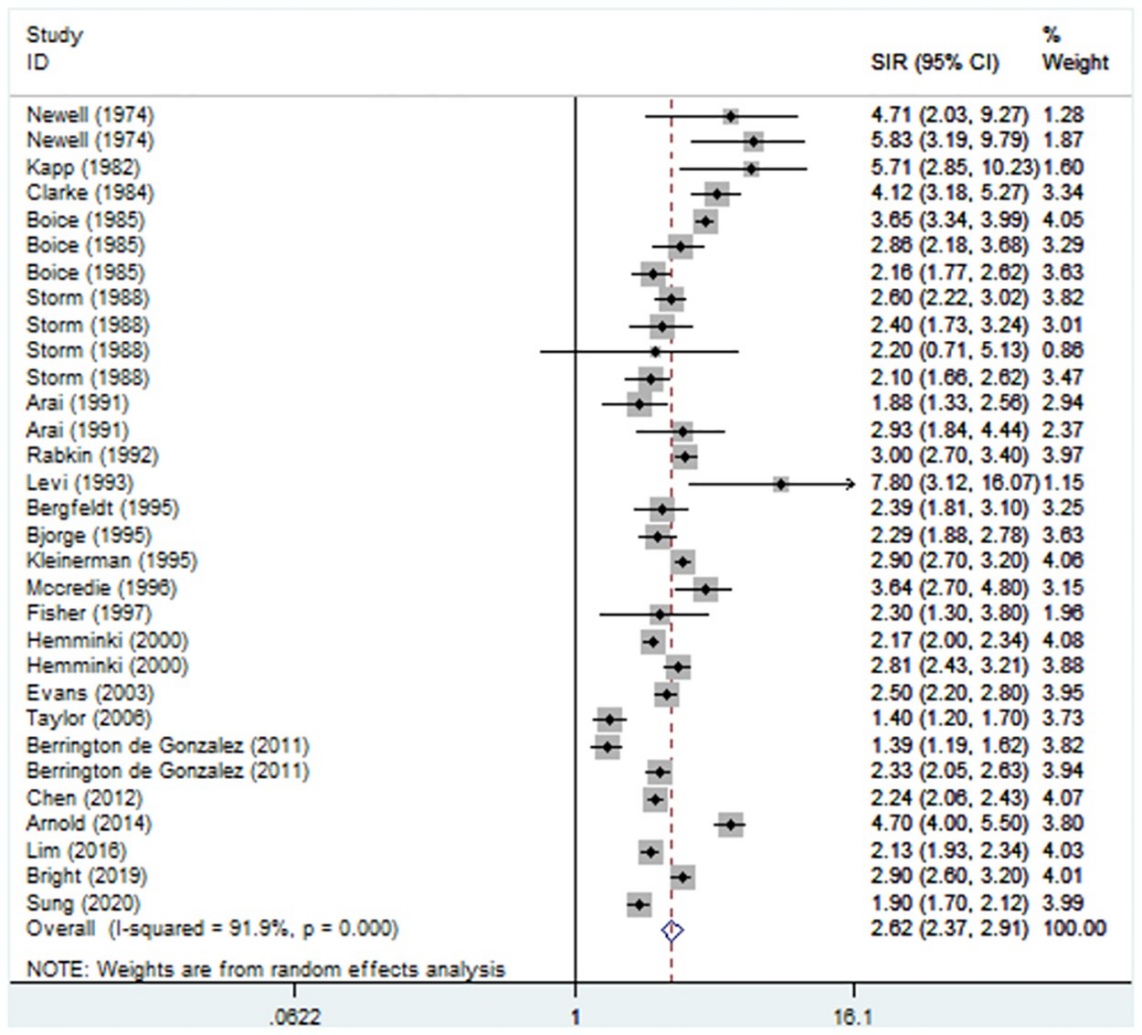
The risk for subsequent primary lung cancer after cervical cancer.

After combining the 22 included studies, the pooled results indicated a significantly increased risk of developing subsequent primary lung cancer among cervical cancer patients (SIR = 2.63, 95% CI: 2.37–2.91, P<0.001; I^2^ = 91.9%, P<0.001) (Fig 2).

Subgroup analysis based on the follow-up period revealed an increased risk throughout the follow-up period (<5 years: SIR = 3.03, 95% CI: 1.83–5.00, P<0.001; ≥5 years: SIR = 2.37, 95% CI: 2.03–2.77, P<0.001) (S1A and S1B Fig). In addition, subgroup analysis stratified by age indicated that cervical cancer patients who were older or younger than 50 years old had an increased risk of developing lung cancer (SIR = 3.27, 95% CI: 1.99–5.40, P<0.001; SIR = 3.94, 95% CI: 3.27–4.76, P<0.001) (S2A and S2B Fig). Furthermore, the degree of malignancy did not affect the risk for subsequent primary lung cancer among cervical cancer patients (invasive: SIR = 2.44, 95% CI: 1.87–3.18, P<0.001; in situ: SIR = 2.00, 95% CI: 1.70–2.36, P<0.001) (S3A and S3B Fig). Subgroup analysis stratified by publication year (before or after 2000) also revealed an increased risk of lung cancer in cervical cancer patients (Table 2).

**Table 2:** Results of meta-analysis.

	No. of studies	SIR	95% CI	P value	I^2^ [%]	P_heterogeneity_
Overall	22 [10, 18–38]	2.63	2.37–2.91	<0.001	91.9	<0.001
Follow-up period						
<5 years	6 [10, 19, 21, 26, 27, 35]	3.03	1.83–5.00	<0.001	96.4	<0.001
≥5 years	8 [10, 19, 21, 27, 28, 31, 35, 37]	2.37	2.03–2.77	<0.001	90.4	<0.001
Age						
Age≥50-year-old	3 [10, 35, 36]	3.27	1.99–5.40	<0.001	97.3	<0.001
Age<50-year-old	3 [10, 35, 36]	3.94	3.27–4.76	<0.001	65.4	0.056
Degree of malignancy						
Invasive	14 [10, 19–22, 24, 28, 30–32, 34–36, 38]	2.44	1.87–3.18	<0.001	99.1	<0.001
In situ	5 [21, 22, 27, 31, 33]	2.00	1.70–2.36	<0.001	77.2	0.001

SIR: standard incidence ratio; CI: confidence interval.

### Sensitivity analysis and publication bias

**Fig 3:**
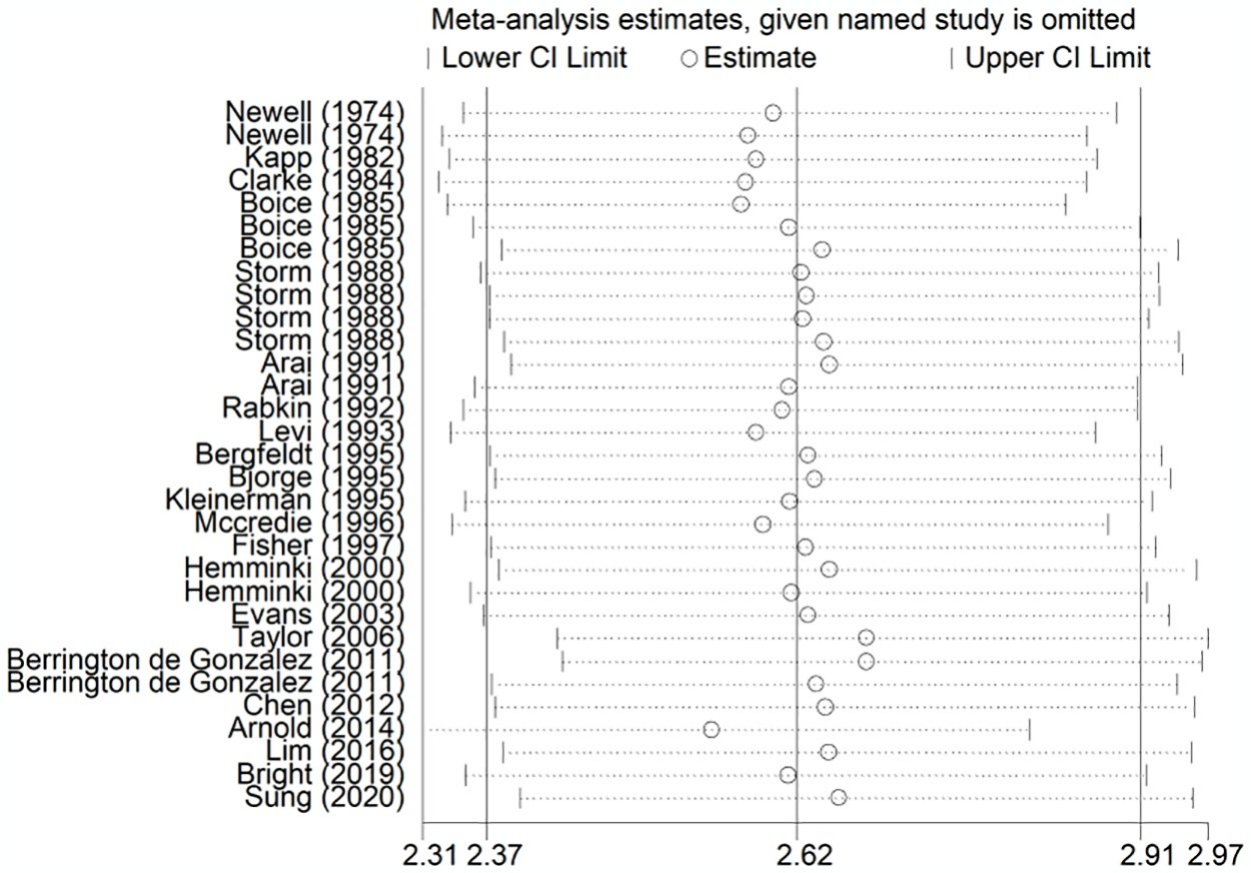
Sensitivity analysis for the risk of developing subsequent primary lung cancer after cervical cancer.

**Fig 4:**
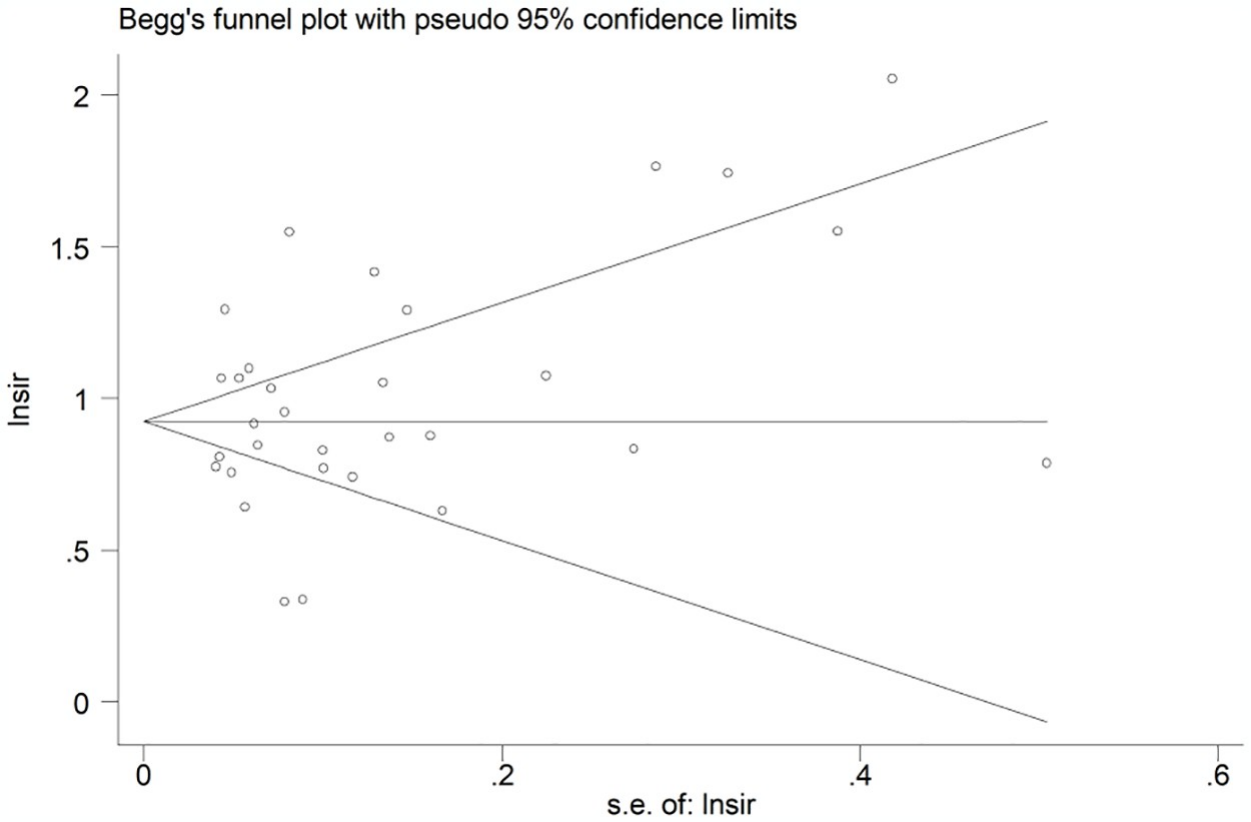
Begg’s plot.

The sensitivity analysis was conducted by excluding each study at one time and showed that the results of this meta-analysis were stable and reliable (Fig 3). In addition, Begg’s funnel plot was symmetrical (Fig 4), and the P value of Egger’s test was 0.501, which indicated that no significant heterogeneity was observed in the current meta-analysis.

## Discussion

The current meta-analysis demonstrated that patients with cervical cancer have a significantly greater risk of developing subsequent primary lung cancer than does the general population. Moreover, subgroup analysis based on follow-up time, age and degree of malignancy showed similar results, and cervical carcinoma patients were indeed more likely to develop lung cancer than the general population. However, due to the high heterogeneity and other limitations, more high-quality prospective studies are still needed to verify our findings.

Based on the current relevant evidence, there are several possible explanations for this phenomenon. First, it is well known that HPV infection is one of the most important causes of cervical cancer, especially high-risk genotypes such as HPV 16 and 18 [39]. Interestingly, in the meta-analysis conducted by Karnosky et al., lung cancer patients showed a weighted overall 4.7-fold (95% CI: 2.7–8.4, P<0.001) increase in the HPV infection rate compared to that of healthy people, especially in squamous cell carcinoma patients, and HPV genotypes 16 and 18 were also proven to be the most prevalent high-risk genotypes [40]. Zhai et al. demonstrated that HPV infection was significantly associated with the incidence of lung cancer [odds ratio (OR) = 5.67, 95% CI: 3.09–10.40, P<0.001], especially lung squamous cell carcinoma (OR = 9.78, 95% CI: 6.28–15.22, P<0.001) after reviewing 1,094 cases and 484 noncancer controls, and HPV16/18 infection was associated with a slightly greater risk for lung cancer (OR = 6.02, 95% CI: 3.22–11.28, P<0.001) [41]. In addition, an increasing number of studies have shown that HPV plays a role in the carcinogenesis, development and progression of lung cancer through several signaling pathways, such as the PI3K/Akt/HIF-1α pathway [42–44]. Second, smoking is another important risk factor for cervical carcinoma [45]. Although the proportion of smokers in women is relatively low, second-hand smoke (SHS) and third-hand smoke (THS) are major public health problems for women. Wen et al. verified that daily SHS and THS exposure were strongly related to increased risks for cervical cancer [hazard ratio (HR) = 1.22, 95% CI = 1.06–1.42; HR = 1.24, 95% CI = 1.03–1.49] [46]. Moreover, longer exposure to SHS and THS led to greater risks (p for trend: 0.006 and 0.035, respectively) [46]. Third, the occurrence of cervical carcinoma is also associated with early marriage, early childbearing, premature sex and multiple births [47–50]. These parameters could increase estrogen levels to some extent, and a number of studies have indicated that estrogen is closely related to the occurrence and development of lung cancer [51–53].

In our study, we were unable to identify the influencing factors for subsequent primary lung cancer due to sufficient data reported in the literature in the form of a meta-analysis. However, a few studies have investigated this topic. Qian et al. reported that advanced age, black race and radiotherapy (all P<0.05) were risk factors for second primary lung cancer in cervical cancer patients after enrolling 15,358 single cervical cancer patients and 451 lung cancer patients with previous cervical cancer [54]. In the study by Arnold et al., advanced age was also reported to be a risk factor for subsequent lung cancer, although this finding is not consistent with the results of subgroup analysis based on age (≥50 years old vs. <50 years old: 3.27 vs. 3.94 for SIR) [36]. Furthermore, Chaturvedi et al. revealed that the increased risk for subsequent primary lung cancer in patients with squamous cell carcinoma of the cervix was more obvious than that in patients with adenocarcinoma of the cervix (SIR: 2.69 vs. 2.18, P = 0.026) [55]. Overall, more relevant studies are urgently needed to identify influencing factors for subsequent primary lung cancer among cervical cancer patients, which would help with the clinical management and intervention of the risk of lung cancer after treatment for cervical cancer.

There are several limitations in this meta-analysis. First, all included studies were retrospective, which might cause bias. Second, due to insufficient information, we were unable to conduct additional subgroup analyses based on the treatment of cervical carcinoma and other parameters or to identify the influencing factors for subsequent primary lung cancer among cervical cancer patients. Third, significant heterogeneity was observed in our study, but we did not find the main sources of heterogeneity according to the results. Fourth, in the study by Levi et al., the specific number of cervical cancer patients was not provided, and 34,615 was the total sample size of this study. Fifth, most cervical carcinoma patients in the included studies were diagnosed before 2000, and the current treatment methods and overall conditions of women are quite different from those before 2000.

## Conclusion

Compared to the general population, cervical cancer patients are much more likely to develop subsequent primary lung cancer despite their age, follow-up time and degree of malignancy. However, more high-quality prospective studies are still needed to verify our findings.

## Supporting information

S1 Fig A. The risk for subsequent primary lung cancer among cervical cancer patients who were follow-up for less than 5 years. B. The risk for subsequent primary lung cancer among cervical cancer patients who were follow-up for more than 5 years.
(PDF)

S2 Fig A. The risk for subsequent primary lung cancer among cervical cancer patients who were older than 50-year-old. B. The risk for subsequent primary lung cancer among cervical cancer patients who were younger than 50-year-old.
(PDF)

S3 Fig A. The risk for subsequent primary lung cancer among invasive cervical cancer patients. B. The risk for subsequent primary lung cancer among patients with carcinoma in situ of the cervix.
(PDF)

## Abbreviations

CI: confidence interval
HPV: human papillomavirus
HR: hazard ratio
NOS: Newcastle Ottawa scale
OR: odds ratio
SHS: second-hand smoke
SIR: standard incidence ratio
THS: third-hand smoke
