# Supplementary material for: The risk for subsequent primary lung cancer after cervical carcinoma: A quantitative analysis based on 864,627 cases
Source: PLoS One. 2024 Jun 24;19(6):e0305670. doi: 10.1371/journal.pone.0305670 (PMC11195986; doi:10.1371/journal.pone.0305670)

Supplementary figure 1A. The risk for subsequent primary lung cancer among cervical cancer patients who were follow-up for less than 5 years.

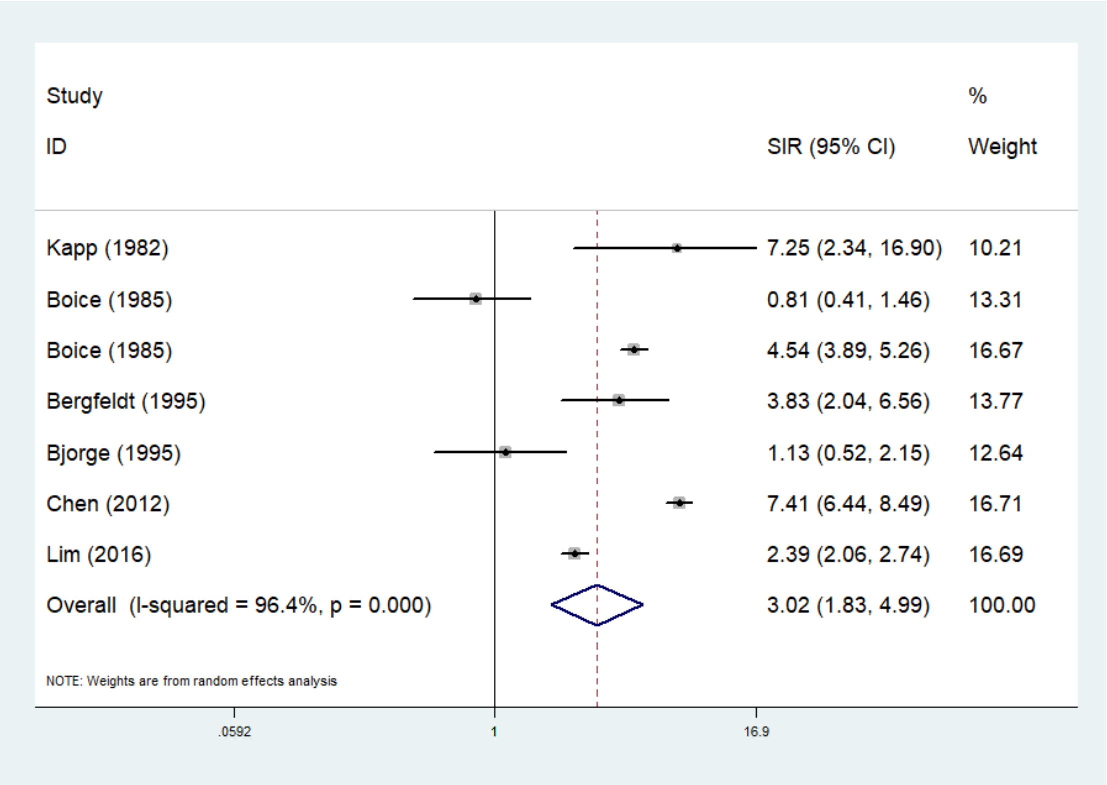

Supplementary figure 1B. The risk for subsequent primary lung cancer among cervical cancer patients who were follow-up for more than 5 years.

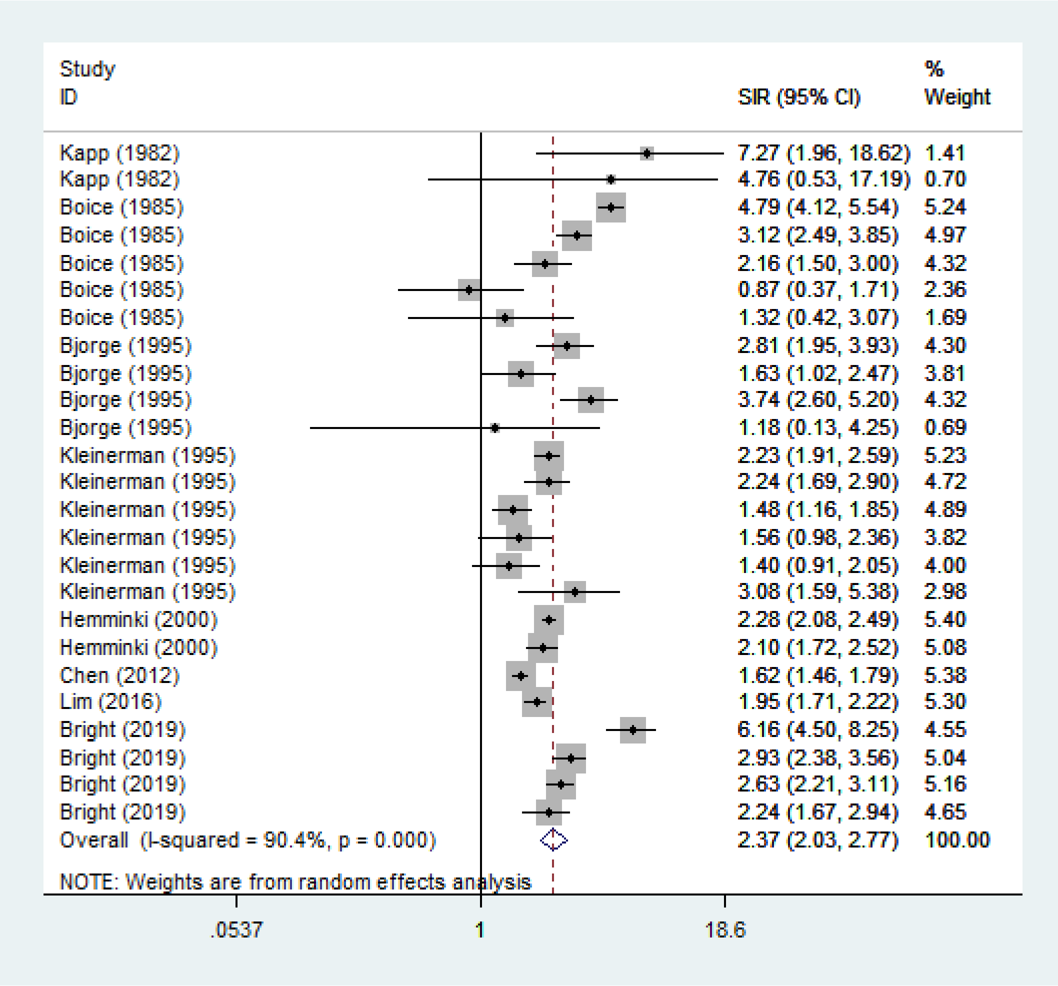

Supplement: S1 Fig — A. The risk for subsequent primary lung cancer among cervical cancer patients who were follow-up for less than 5 years. B. The risk for subsequent primary lung cancer among cervical cancer patients who were follow-up for more than 5 years. (PDF) [file pone.0305670.s001.pdf]
