# Supplementary material for: The risk for subsequent primary lung cancer after cervical carcinoma: A quantitative analysis based on 864,627 cases
Source: PLoS One. 2024 Jun 24;19(6):e0305670. doi: 10.1371/journal.pone.0305670 (PMC11195986; doi:10.1371/journal.pone.0305670)

Supplementary figure 2A. The risk for subsequent primary lung cancer among cervical cancer patients who were older than 50-year-old.

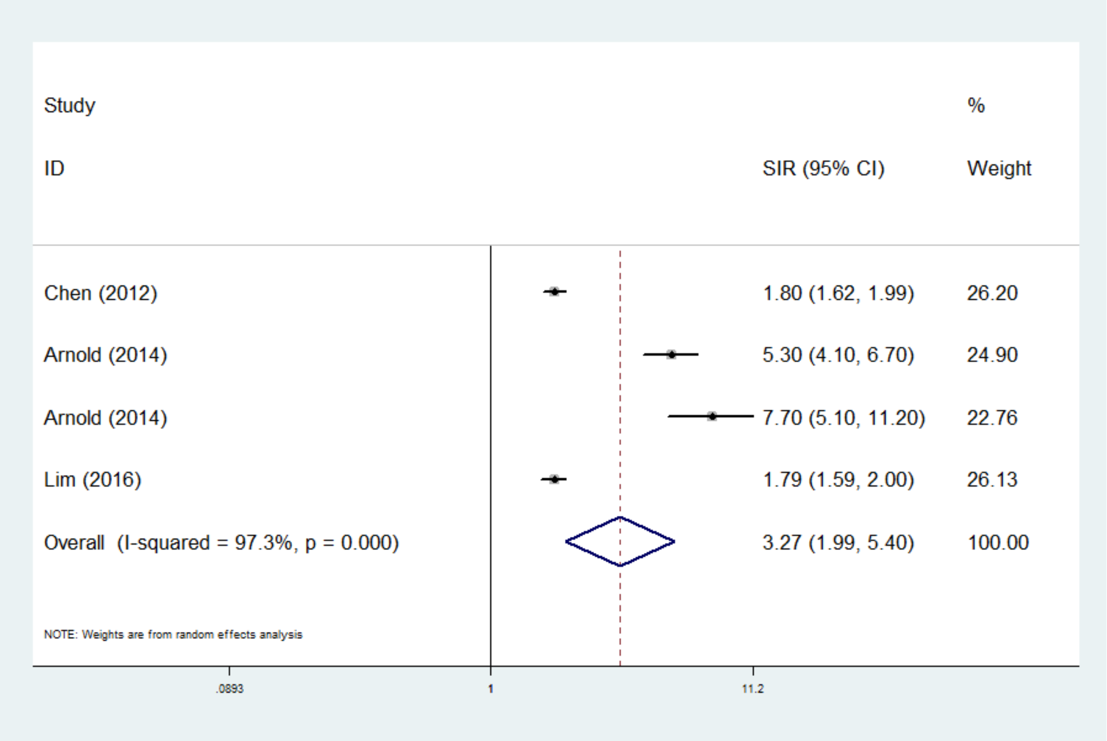

Supplementary figure 2B. The risk for subsequent primary lung cancer among cervical cancer patients who were younger than 50-year-old.

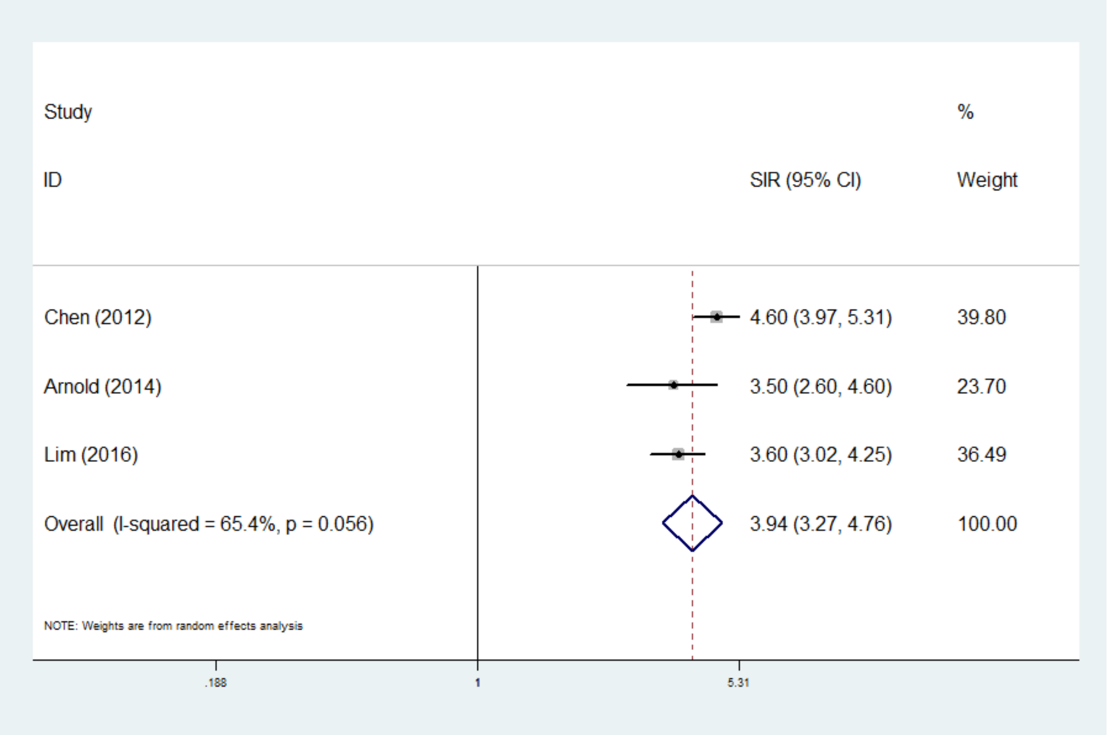

Supplement: S2 Fig — A. The risk for subsequent primary lung cancer among cervical cancer patients who were older than 50-year-old. B. The risk for subsequent primary lung cancer among cervical cancer patients who were younger than 50-year-old. (PDF) [file pone.0305670.s002.pdf]
