# Supplementary material for: The risk for subsequent primary lung cancer after cervical carcinoma: A quantitative analysis based on 864,627 cases
Source: PLoS One. 2024 Jun 24;19(6):e0305670. doi: 10.1371/journal.pone.0305670 (PMC11195986; doi:10.1371/journal.pone.0305670)

Supplementary figure 3A. The risk for subsequent primary lung cancer among invasive cervical cancer patients.

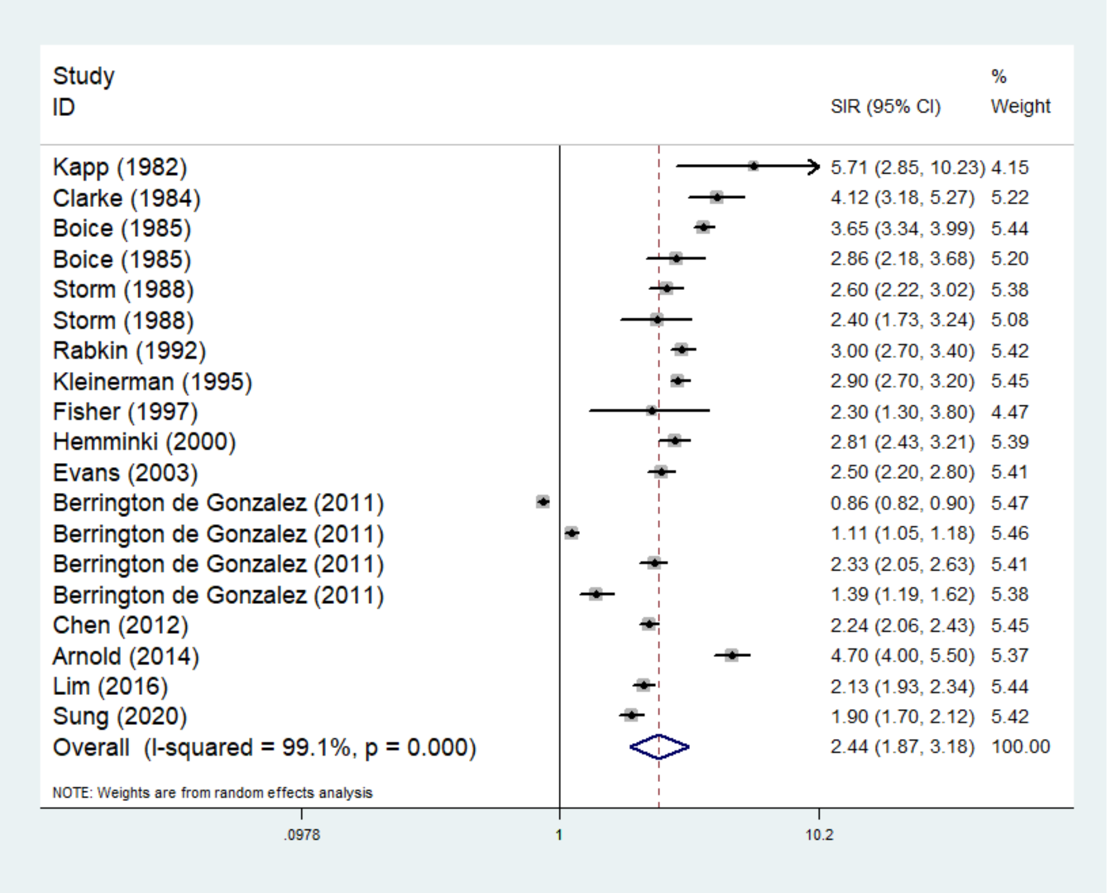

Supplementary figure 3B. The risk for subsequent primary lung cancer among patients with carcinoma in situ of the cervix.

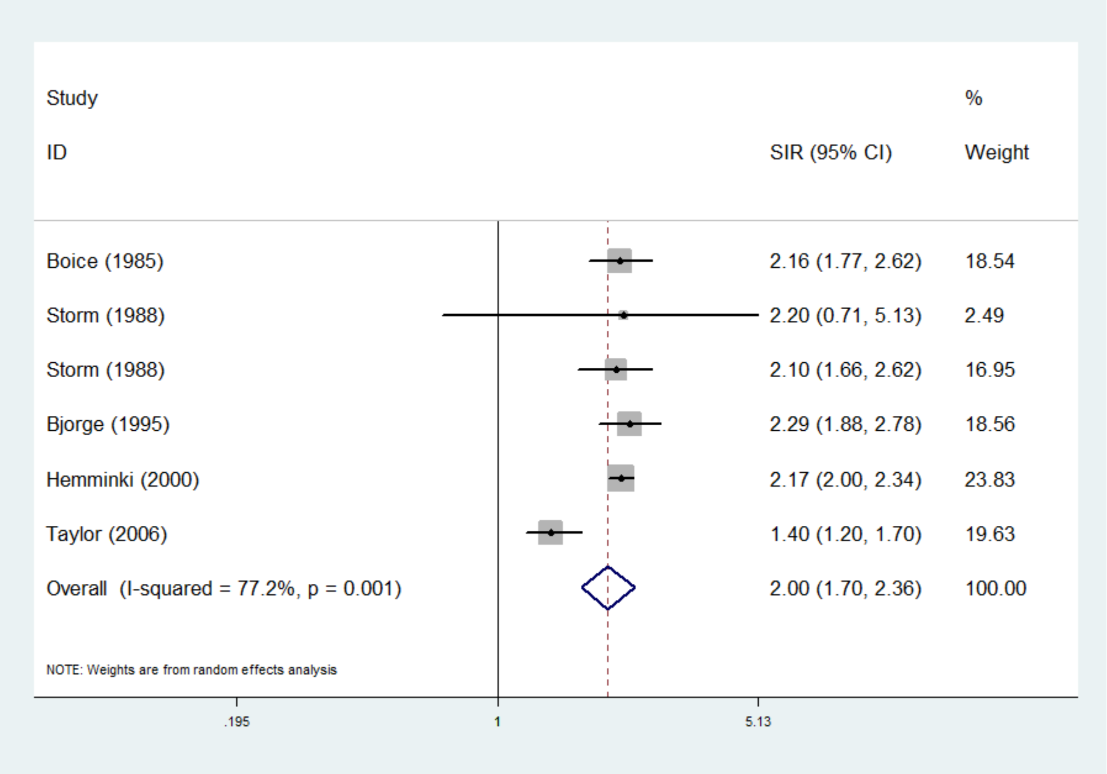

Supplement: S3 Fig — A. The risk for subsequent primary lung cancer among invasive cervical cancer patients. B. The risk for subsequent primary lung cancer among patients with carcinoma in situ of the cervix. (PDF) [file pone.0305670.s003.pdf]
